# A Novel Clinical Perspective on New Masses after Lead Extraction (Ghosts) by Means of Intracardiac Echocardiography

**DOI:** 10.3390/jcm9082571

**Published:** 2020-08-08

**Authors:** Carlo Caiati, Giovanni Luzzi, Paolo Pollice, Stefano Favale, Mario Erminio Lepera

**Affiliations:** Unit of Cardiovascular Diseases, Department of Emergency and Organ Transplantation, University of Bari, 70123 Bari, Italy; luzzi.giovanni@yahoo.it (G.L.); paolo.pollice@yahoo.it (P.P.); stefano.favale@uniba.it (S.F.); marioerminio.lepera@uniba.it (M.E.L.)

**Keywords:** ghosts, intracardiac echocardiography, lead infection, lead encapsulation

## Abstract

Background: A lead-reactive fibrous capsule (FC) identified by ultrasounds as an atrial or ventricular lead thickness of more than 1 mm above the vendor-declared lead diameter (TL) and its local fibrotic attachment to the cardiac wall (FAC) have never been investigated in vivo, so their relationship with post-extraction masses (ghost) is not known. Methods: Intracardiac echocardiography (ICE) was performed twice during the same extraction procedure in 40 consecutive patients: before and immediately after infected lead extraction Results: The ghost detection rate was high: 60% (24/40 patients); ICE could identify both TL and FAC, TL being noted in 25/40 (62%) patients and FAC in 12/40 patients (30%). Both TL and FAC were significantly associated with ghosts (*p* < 0.001 and *p* = 0.002, respectively), but TL had a higher prediction power. The specificity was similar: 94% (15/16) and 100% (16/16), respectively, but TL showed a much higher sensitivity: 100%, (24/24) vs 50% (12/24) (*p* = 0.016). The ghost group did not show a higher event rate in the follow-up (mean follow-up time = 20 ± 17 months). Conclusion: ICE is able to evaluate both TL and FAC in vivo; ghosts are mostly benign remnants of fibrotic lead capsule cut off during extraction and retained inside the heart by FAC.

## 1. Introduction

Infection of cardiac implantable electronic devices (CIEDs)—such as permanent pacemakers and implantable cardioverter defibrillators—is a severe occurrence associated with high mortality [1]. The complete removal of CIED is the recommended treatment for patients with an established cardiovascular implantable electronic device infection [2].

After lead extraction, new mass lesions may ensue, in 8%, 14% and 19% of cases [3,4,5], in the intracardiac lead route attached to the right cavity endocardium.

Only three studies in the literature have addressed the problem of ghosts in a large number of patients [3,4,5], but in these 3 studies the nature of these new masses was not clearly demonstrated. The poor knowledge of the nature of these masses causes uncertainty as regards their prognostic impact and hence the clinical management of patients. Reliable predictors of ghost formation are lacking. Pathology studies both in humans [6,7] and animals [8] have previously demonstrated that implanted leads, especially when their insulation structure is damaged [9], trigger a thrombotic reaction on the lead and also on the nearby cardiac endocardium, which is eroded by the lead [8]. Ultimately, the thrombotic reaction brings about lead fibrotic encapsulation and adherence to the cardiac wall [10,11]. This resulting fibrotic lead encapsulation and adhesion could be strongly connected to post-extraction masses, as previously speculated [12], but never before directly investigated in vivo by an imaging approach. In our preliminary experience (unpublished), we found that the thickness of the newly implanted lead can be correctly assessed by transthoracic echocardiography (TTE), so we hypothesized first, that a highly sensitive imaging technique like intracardiac echocardiography (ICE) [13] has the potential to investigate thrombotic/fibrotic reactions involving the leads, in particular assessing lead thickness. Second, that this ICE-detected fibrotic reaction could be a strong predictor of “ghost” masses. Third, that transesophageal echocardiographic follow-up should shed light on the composition of these masses, since after the elimination of the thrombogenic stimulus, the acute thrombotic stratification, should disappear in a couple of weeks [14], whereas the fibrosis should persist over time.

In order to verify these hypotheses, we prospectively enrolled 40 consecutive patients scheduled for transvenous infected lead extraction. They underwent periprocedural ICE before and after extraction and then clinical and transesophageal echocardiographic follow-up.

## 2. Methods

In this exploratory, unicentric, prospective study we enrolled 40 patients from January 2014 to September 2017, referred to our department for lead(s) extraction due to infective endocarditis. All data concerning the clinical and microbiological status of the patients were recorded before the extraction procedure.

All enrolled patients underwent both intracardiac echocardiography (ICE) and transesophageal echocardiography (TEE). ICE was performed twice during the extraction procedure: before extraction in order to gain a pre-extraction picture of the catheter and again immediately after extraction. TEE was only performed during follow-up (25 ± 5 days). All patients gave written informed consent to enter the study. (study protocol approved by “Comitato Etico Indipendente, Azienda Ospedaliera-Universitaria Policlinico, Bari, IT,” code of approval 4330.)

### 2.1. Clinical Management

During hospitalization, all patients were administered antibiotic therapy; wound swab culture and blood cultures were performed before (preferably in wash-out from antibiotic therapy) and after the extraction; lead(s) tip culture was performed after the procedure.

In the pre-extraction phase, cardiac device-related infective endocarditis (CDRIE) was defined accordingly to the previously validated, recently updated criteria [1]; thus, definite endocarditis was considered if at least two of the following major criteria were present: lead vegetations, positive blood culture, local device infection and also a positive tip culture, but only in the absence of local device infection [15]; only 1 of these major criteria in the absence of at least 3 minor criteria (fever, septic pulmonary embolism, pulmonary infiltrates, arthralgia, spondylitis, etc.) was considered possible endocarditis [16]; major bacteriological criteria were positive blood cultures for typical endocardial pathogens or persistently positive for a microorganism consistent with infective endocarditis [15,16]. Local device infection was clinically defined as local signs of inflammation, erythema, wound dehiscence or erosion.

### 2.2. Intracardiac Echocardiography

We used catheters (8 Fr, Acunav^TM^ Siemens, Munich, Germany) equipped with a linear phased array multifrequency (5.5 to 10 MHz) steerable transducer with 4 steering directions: anterior/posterior and left/right deflection of the catheter. The transducer produces a longitudinal scanning plane with a 90° sector image and has a tissue penetration capability of 15 mm. It was connected to a Siemens Acuson P50 portable ultrasound system (Siemens, Munich, Germany) or an Acuson Sequoia 512 Ultrasound System (Siemens, Munich, Germany).

#### 2.2.1. Before Extraction

The AcuNav catheter (length of 90 cm) was inserted into a femoral vein and, under fluoroscopy guidance, advanced through the inferior cava vein up to the superior vena cava; then, images were obtained first at the level of the superior vena cava and then, after appropriate withdrawal, at the level of the right atrium. At this level it was rotated and tilted up and down in order to visualize the catheter(s) and all atrial segments, including the right appendage; in rare cases it was then curved and advanced into the right ventricle to better explore beneath the tricuspid valve; in all these regions ICE allowed accurate visualization of the leads and of the right cardiac structures, as previously described [13].

To allow the extraction procedure the AcuNav was withdrawn down to the inferior vena cava.

#### 2.2.2. After the Extraction

The catheter was repositioned again up to the superior vena cava and the imaging sequence as described before was repeated.

### 2.3. Extraction Procedure

Before the procedure, pacemaker-dependent patients were equipped with an endovascular temporary stimulator positioned through a femoral vein approach in the right ventricle apex. The leads extraction procedure was performed first by surgical pocket isolation of the extravascular portion of the leads—cutting the proximal component and separate collection in a sterile container of strips of pocket tissue for bacteriological evaluation by culture tests. Subsequently, extraction of the intravascular and intracardiac portion was performed by manual technique using the polypropylene dilators COOK PLEBES^®^ liberator (Cook Medical, Bloomington, IN, USA), rotation and counter-rotation movements until complete detachment of the leads. At the end of removal of each individual lead, its distal component was collected in a separate sterile container for bacteriological evaluation by culture test [17]. The extraction procedure was successfully performed in all patients. Data concerning lead- and procedure-related factors such as the number of leads removed, the type of device (pacemaker or implantable cardioverter defibrillator), time of first implantation, presence of coronary sinus lead, catheter loop(s), were collected.

Most of our patients (almost 70%) had a transvenous reimplantation some time (3–4 weeks) after the extraction. In a minority, however, when the patient was not pacing dependent, no more reimplantation was done.

### 2.4. Transesophageal Echocardiography

Transesophageal echocardiography (TEE) was performed after the extraction procedure (within one month) using commercially available equipment (Philips IE33, Eindhoven, Holland, ultrasound system) connected to a multiplane transducer (Philips X7-2t, 5 MHz transducer). Local anesthesia was applied to the hypopharynx by spraying it with xylocaine and all patients were sedated with benzodiazepines. The endoscope was introduced into the esophagus with the patient in left lateral position. A complete scan of the entire tricuspid valve and the leads was performed, tilting and alternately withdrawing and advancing the tip of the endoscope. In particular, the more specific TEE approaches for lead imaging before extraction were: bicaval TEE approach for the intra-caval and intra-atrial course (including the right atrial appendage course); cardial for the intracoronary sinus lead; and both transgastric and mid-esophageal 4-chamber for the intraventricular course.

### 2.5. End Points

In the pre-extraction phase, we specifically aimed to visualize the mass-related endocarditis, but also, for the first time, specific echographic features never before reported in literature: the fibrosis encapsulating the leads and fixing it to the cardiac wall. We assessed the presence of ghosts in the post-extraction phase with ICE and in the follow-up with TEE. Endocarditis vegetation was defined as an oscillating intracardiac mass on the electrode lead(s), cardiac valve leaflets or endocardial surface, confirmed by imaging in more than 1 echographic plane, in case of valve or lead infection identified by positive blood or lead-tip cultures [16,18,19]. We collected information about the localization, shape and size of the vegetation and in cases of multiple masses, the largest was used for analysis.

Fibrosis encapsulating leads was considered if a thickened lead (TL) was observed. To preliminarily evaluate the potential of ultrasound in assessing this parameter we carried out a preliminary transthoracic study: six newly implanted patients were evaluated by TTE (subcostal approach) the day after the implantation to measure lead ventricular thickness and to compare this measured value with the vendor-declared thickness. We found that the limits of agreement were very narrow (lower than 1 mm): limits of agreement = 0.58 (95CI 0.19–0.98) mm and −0.22 (95CI −0.6–−0.2).

Hence, considering the higher lateral and axial resolution of ICE we decided that a 1 mm greater thickness than the vendor-declared value should very reliably indicate an abnormal lead thickness due to lead encapsulation. Hence, TL was defined at ICE as either atrial or ventricular lead measured thickness of more than 1 mm above the vendor-declared lead diameter. The transverse measurement of the lead was performed leading edge-to-leading edge by calipers in various planes, preferring visualization of the lead with the axial resolution of the ultrasound beam. The coil inside the lead had the typical helicoid echo-appearance (alternate strips of bright and dark echoes) as the coil includes two parallel electrically conductive strips separated by a narrow gap. For ventricular leads both the intra-atrial and intra-ventricular course of the leads was assessed: in all cases the maximal measure was considered. In addition, we tested intra- and interoperator variability in 10 randomly chosen cases: the same measurement was taken by two different operators to test inter-observer variability and then it was re-evaluated by one of the two, 10 days later.

Lead fibrotic attachment to the cardiac wall (FAC) was defined at ICE either as sleeve-like dense echoes involving the leads and extending to the surrounding structures (tricuspid valve, atrial and ventricular wall) with loss of independent lead motion or when a thickened lead was adherent to the atrial/ventricular endocardium or the tricuspid valve and followed the cardiac motion of that structure, having lost its independent motion.

Ghosts were defined as new, post-removal, mobile masses visualized by ultrasound (ICE and/or TEE postprocedural) [3]; we collected information about the localization, shape and sizes (maximal and minimal) of ghosts and we investigated the association with CDRIE, positive blood and lead culture after extraction, number of leads removed and age of the leads.

### 2.6. Clinical Follow-up

To better understand the real prognostic impact of ghosts, all patients underwent clinical follow-up (mean follow-up time = 20 ± 17 months); the events investigated were death, pulmonary embolism and infiltrates, new infections, fever.

### 2.7. Statistical Analysis

Continuous variables were expressed as arithmetic means and standard deviation. Dichotomous variables were expressed as number and percentage. The Shapiro-Wilk test was used to test for normal data distribution.

Differences between groups (ghost and no ghost group and lead fibrosis and no lead fibrosis group) were analyzed using Student’s t-test for continuous variables and either Yates’ χ^2^ test or Fisher’s exact test, as appropriate, for dichotomous variables and phi coefficient for calculating effect size statistics (0.10, 0.30 and 0. 50 indicate small, medium and large effect, respectively). The odds ratio (OR) and 95% confidence interval were calculated according to Altman [20].

Receiver operating characteristic curve was used to assess the diagnostic accuracy and the best cutoff of TL measurement versus the dichotomic ghost variable.

White blood cells counts versus the 3 groups of lead fibrosis were tested by linear contrast analysis using between-groups one-way ANOVA and Eta squared was calculated for the effect size with ANOVA (0.01 indicates small effect, 0.06 medium effect and 0.138 large effect).

The Bland-Altman method was used for assessing the limits of agreement and the coefficient of variability between the repeated measurements of lead thickness [21].

A multivariate analysis was carried out with a Binary Logistic model using both forward and backward stepwise methods, in order to explore any interaction effect among the prognostic variables.

For each analysis, the significance level was set at α = 0.05, that is pertinent also in cases of small samples and robust in rejecting null hypotheses.

Statistical calculations were performed using both IBM SPSS statistics version 23, (IBM Corp, Armonk, NY, USA).

## 3. Results

General demographic and clinical data of the whole study group are reported in Table 1.

In our series 10 patients (25%) had possible endocarditis (only one major criterion) while 30 (75%) had definitive endocarditis with either 2 (22 patients (55%)) or 3 (8 patients (20%)) major criteria. Local device infection was present in most these patients: 33 patients (82.5%).

### 3.1. Pre-extraction

ICE showed an endocarditis mass lesion in 27/40 patients (67%), being single (23 patients) or multiple floating masses (3 patients) with a max dimension of 8 ± 4 mm, attached mainly to the leads (either ventricular in 20 patients (74%), atrial in 5 patients (18%) or coronary sinus lead in 2 patients (7%), but also in 3 patients (11%) to the right atrial walls.

**Figure 1:**
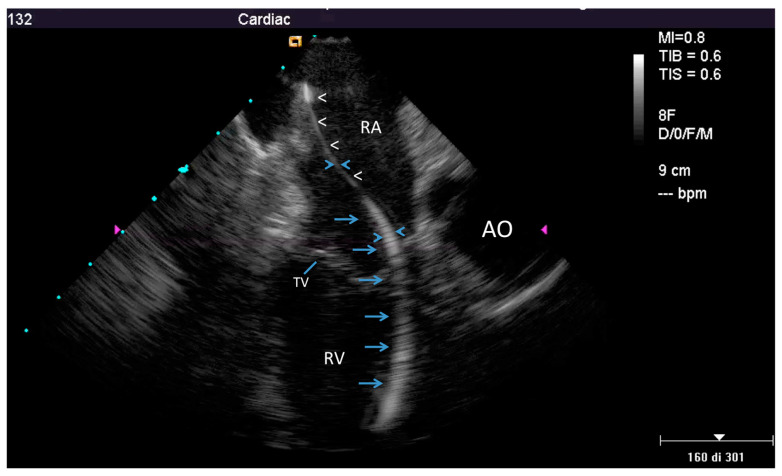
Example of a partially thickened lead at intracardiac echocardiography (ICE). Home view showing normal thickness of the proximal part of the lead in the right atrium (white arrow head), but evident thickening in the distal atrial part and in its ventricular course (≥4 mm, blue arrows); AO—aorta; RA—right atrium; RV—right ventricle; TV—tricuspid valve.

**Figure 2:**
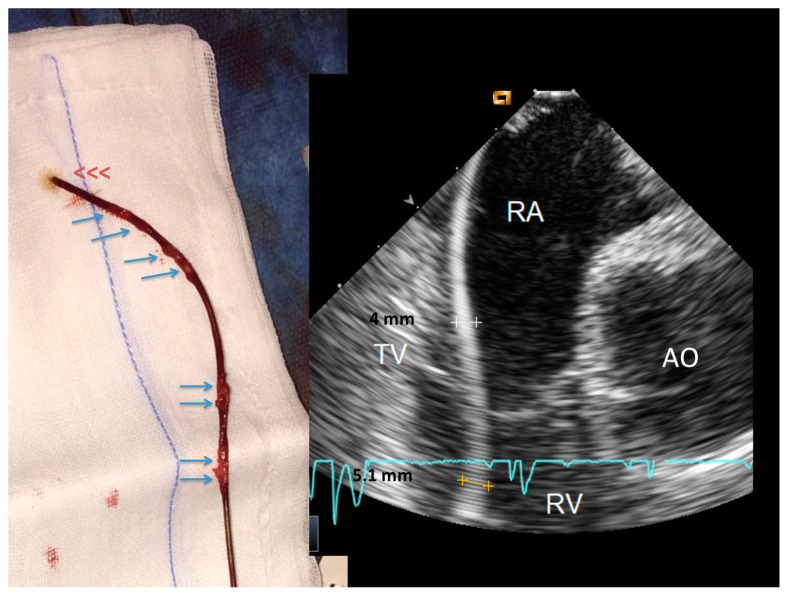
Side-by-side, a thickened lead at ICE and the same lead at visual inspection immediately after extraction. The ventricular lead (right side) is uniformly thickened at ICE. After extraction, the lead (left side) shows remnants of sheathing fibrosis in both the ventricular and atrial parts of the lead (arrows). Abbreviations as in Figure 1.

ICE could identify both TL and FAC; TL (thickness ≥ 1 mm than the vendor declared thickness in at least one lead) was noted in 25/40 patients (62%) overall (Figure 1 and Video S1) involving the atrial (2 patients (5%)) or the ventricular (9 patients (22.5%)) or both leads (14 patients (35%)). In particular, 16/33 atrial lead and 23/39 ventricular lead were thickened (uniformly in 3 and partially in 10 patients; max thickness = 4.3 ± 0.5 mm, 95% CI 4.0–4.4). In the remaining 15 (40%) patients no TL was noted (max thickness was of 2.2 ± 0.4 mm, 95% CI 1.9–2.3, very close to the vendor declared diameter of 2.1 ± 0.05 mm, 95% CI 2.07–2.2, *p* = ns). We also assessed, but only qualitatively, the presence of the fibrotic remnants sheathing the extracted lead, visually evaluated after extraction and compared with the ICE pre-extraction assessment. In all patients with TL by ICE we observed a variable residual amount of fibrotic capsule in the distal intracardiac tract of the extracted lead (Figure 2) while in case of normal lead at ICE the distal intra-cardiac part of all extracted leads appeared normal.

Measurement of maximal lead thickness by ICE showed minimal intra and inter-observer variability, the coefficient of repeatability from duplicate measurements being 0.74 mm (95% CI 0.52 to 1.30) and 0.77 mm (95% CI 0.22 to1.32), respectively (Figure S1).

**Figure 3:**
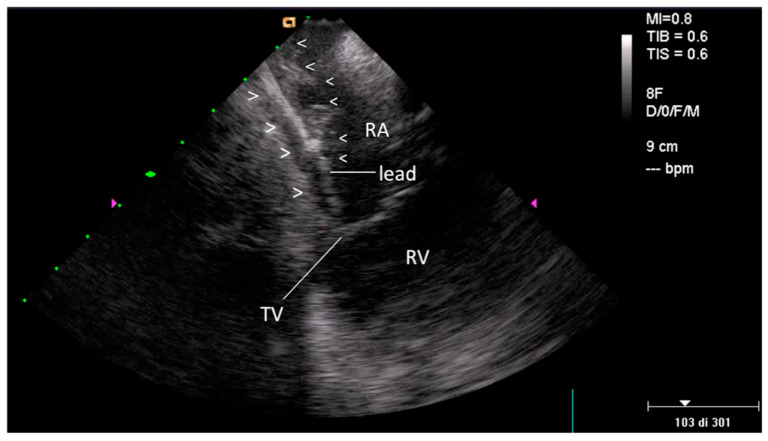
Fibrotic attachment to the cardiac wall (FAC) involving the atrial portion of a ventricular lead. Home view: FAC is visible as dense echoes encasing the atrial portion of a ventricular lead (arrow-heads), fixed to the atrial endocardium with loss of independent lead motion (such findings are better seen in the movie). FAC = lead fibrotic attachment to the cardiac wall; RA—right atrium; RV—right ventricle; TV—tricuspid valve.

On the other hand FAC, always associated with TL, was observed in 12/40 patients (30%): the location of the cardiac lead attachment was mainly in the atrial/auricular chamber (in 7 patients (58%)) (Figure 3 and Video S2), but in rare cases in the ventricular chamber (in 2 patients (16%)), in the superior vena cava near the cardiac outlet (in 1 patient (8%)) or at the level of both right cardiac chambers (in 2 patients (16%)).

**Figure 4:**
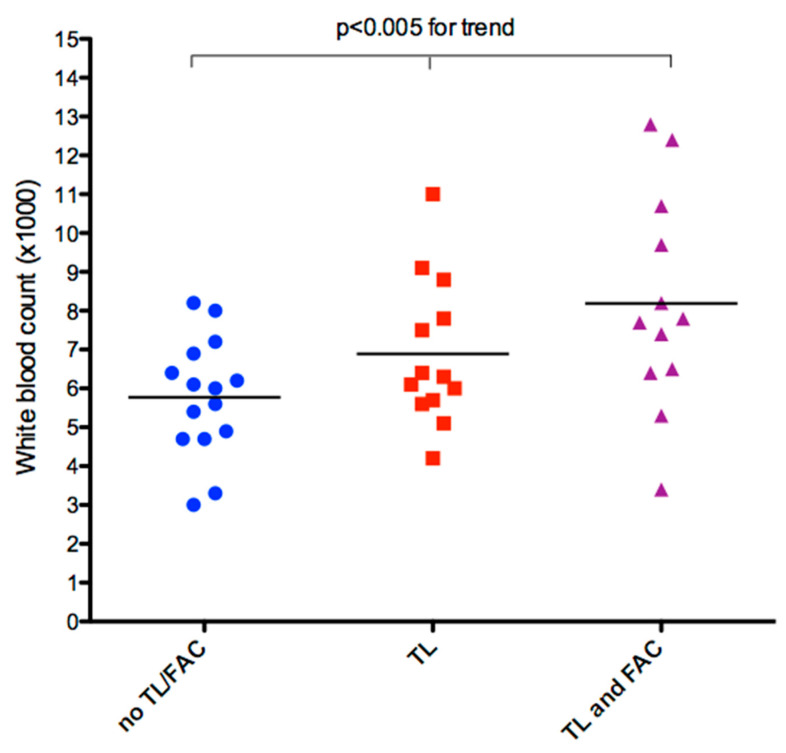
Individual value bar graph of WBC count versus lead fibrosis. There is a significant, progressive mean increase in WBC, with a large effect size all the way up to the major classes of lead fibrosis, as assessed by ultrasound. (F = 9.007, *p* < 0.005 for trend, partial eta squared = 0.196); TL—thickened lead; FAC—fibrosis fixing the lead to the cardiac wall; WBC—white blood cell count.

The presence of TL was significantly associated with higher white cell counts and longer implantation time (Table 2). In addition, the presence in the same patient of both TL and FAC was significantly associated with even higher blood cell count (Figure 4).

Bacteria were isolated in the blood in a minority of patients (13 patients (35%)) while more bacteria were isolated from lead culture (30 patients (75%)) and from infected pocket swabs (in 30 patents (75%)). The isolated microorganisms isolated in the blood were mainly coagulase negative *Staphylococci* (9 patients, (22.5%)) and *Staphylococcus aureus* (5 patients (12.5%)).

### 3.2. Post-extraction ICE

**Figure 5:**
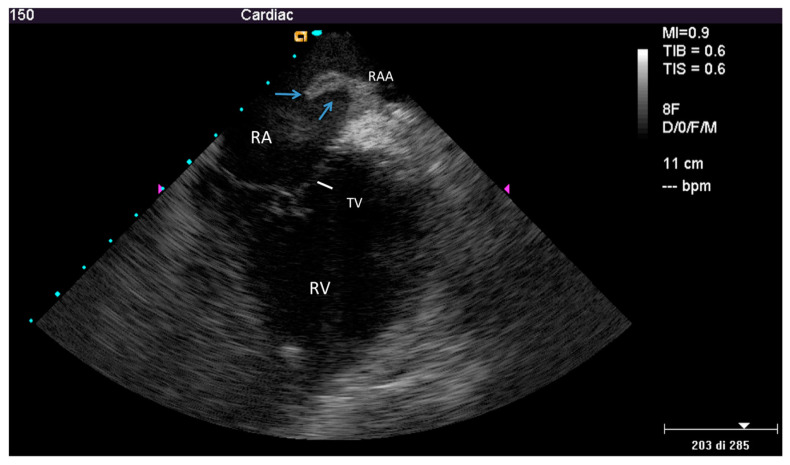
Ghost after lead extraction by ICE. Modified home view by ante-flexion, to gain a close-up of the right ventricle. Mobile hypo-echogenic mass (arrows) in the right atrium attached at the origin of right atrial appendage. In the movie, other smaller floating masses are also seen, attached to the superior vena cava. RAA—right atrial appendage; other abbreviations as in Figure 3.

**Figure 6:**
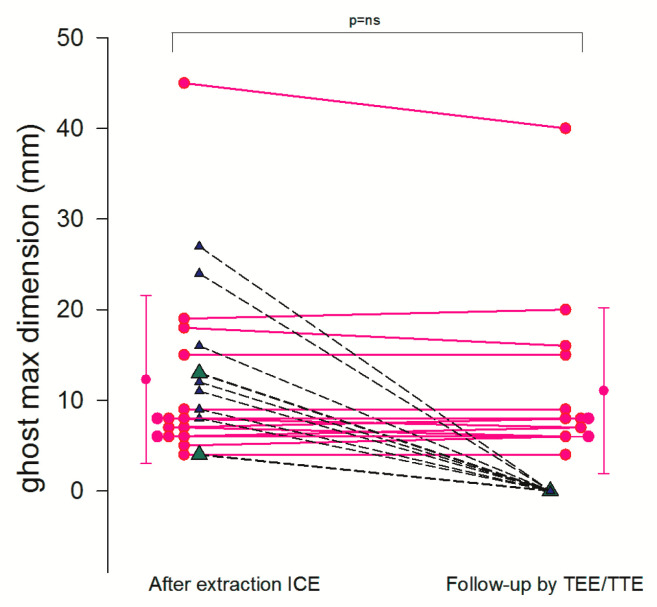
Ghost follow-up at 1 month. Individual value bar graph shows the destiny of the ghost as assessed by ICE (first column) and at 1 month follow-up by TEE (second column). Most of the ghost masses were still there (pink dots connected with solid line), while 7 had disappeared (triangles connected with dashed line). (2 masses, green triangles, were not assessed at follow-up because they were in an inaccessible part of the superior vena cava).

ICE identified new post-extraction masses (ghosts) in a majority of patients (24/40) showing a detection rate of 60% (Figure 5 and Video S3). Maximal and minimal dimension were 12.3 ± 9.3 mm and 3.6 ± 3.2 mm, respectively (Figure 6); the masses were more frequently attached to the right atrial wall/appendage and to the superior vena cava: in 11 patients (46%) and in 10 patients (42%), respectively, but also to the tricuspid valve in 6 patients (24%), right ventricle wall in 1 patients (4%). A single mass was observed in 20 patients (83%) and multiple masses in 4 patients (17%).

**Figure 7:**
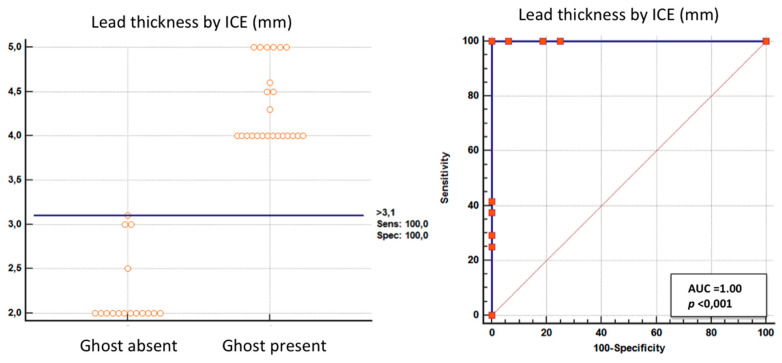
ROC curves. **Left**: bar graph showing the maximum individual measured lead thickness in patients with and without ghosts and the cutoff value (solid horizontal line). **Right**: plot of sensitivity against 1-specificity of the measured lead thickness (ROC curves) in predicting a ghost. A—area under the curve.

Both TL and FAC were significantly associated with ghosts (Table 3) with a very large effect size (*p* < 0.001, phi = 0.94 and *p* = 0.002, phi = 0.53, respectively), but TL had a higher prediction power. In fact, specificity was equally very high for TL and FAC (94% (15/16) and 100% (16/16), respectively), but TL showed a much higher sensitivity: 100%, (24/24) vs 50% (12/24), respectively, (*p* < 0.001). Using the lead thickness cutoff >3.1 mm (bootstrapped 95% confidence interval >3 to >3.1 mm) by ROC analysis we eliminated the only false positive with borderline TL on the basis of our previous criterion of TL (ICE lead thickness >1 mm than the vendor declared lead thickness) (Figure 7).

Infective variables such as vegetations, positive blood cultures before and after extractions were not associated with ghosts (Table 3). Explorative multivariate logistic analysis indicated that the dichotomic TL variable (as assessed by vendor lead thickness comparison) was the only independent predictor of ghosts and correctly classified 97.5% of cases.

In the follow-up by TEE at one month the masses were still there in most patients (15 patients, 62%) with the same max dimension (11.1 ± 9 mm) as immediately after extraction, as visualized by ICE (12.3 ± 9.3 mm, *p* = ns). However, ghost masses had completely gone at 1 month follow-up in 7 patients (37%) (Figure 6).

In the patients without ghosts (16, 40%) no new masses were discovered at the follow-up by TEE.

### 3.3. Prognostic Impact of Ghosts

All patients were followed-up for a mean of 20 ± 17 months. Four patients died, only 1 due to complications probably related to ghosts (ARDS) (Table 4); In addition, 4 other patients developed (in the first month) transient, benign pulmonary infiltrates seen at the chest X-ray. Considering a composite end-point (death plus pulmonary infiltrates) ghosts were not associated with this composite endpoints: the event rate was 20.8% (5/24) in the ghost group and of 18.8% (3/16) in the no ghost group (OR = 1.14, 95% CI 0.23–5.62, *p* = ns).4.

## 4. Discussion

We have demonstrated for the first time that fibrosis involving the lead can be evaluated by ICE and is the major independent predictor of ghost formation so that causation may be inferred. In addition, the follow-up study of ghosts by TEE indicates that these new masses are essentially fibrotic (since they persist unaltered over time in high percentages) although in some cases (37%) fresh thrombus stratification may be prevalent. Finally ghosts per se seem to have neither a bad prognosis (in particular embolism) nor to be specifically connected to new flaring endocarditis episodes in the post-extraction period.

### 4.1. Fibrosis Involving Leads

We believe that a thrombotic-fibrotic reaction toward the leads is a major, initial event that has in general been ignored by imaging studies in vivo [3,22]. Now for the first time this crucial aspect of the leads status, regarding both leads encapsulation and parietal fibrotic adhesion previously only assessed by autoptic studies [7,8,10], can be evaluated in vivo thanks to the spectacular close up of the lead with no ultrasound beam attenuation obtained with ICE [23,24] (Figure 1 and Figure 3). Two major factors trigger the fibrosis that starts with a thrombotic reaction that then becomes organized [7]: one is wear of the outer insulation of the leads (made either of polyether polyurethane or silicone), more evident in the intracardiac part of the leads as confirmed with the stereomicroscope [9] since this is more subjected to hemodynamic forces. The other is lead-induced damage to the parietal endocardium [8]. These are the reasons why the longer the implant lasts, the more extensive the encapsulation [11], since a longer duration (Table 2) drives more wear on the leads, even if the timing of the damage is unpredictable [7].

This leads reaction is also confirmed by the significant positive association found in our study between elevated high white blood cells and progressively increased lead fibrosis as assessed by ICE (Figure 4).

The reaction to the implant may also be modulated by thrombophilic conditions like diabetes and chronic renal diseases [24], although these were not found to be associated with a thickened lead. However, in our group patients had only mild renal disease (Table 1) while diabetes showed a positive, but not statistically significant trend (Table 2).

### 4.2. Nature of the Ghost

Our data very strongly support the connection of the thickened lead and fibrosis to the formation of the post-extraction masses (Table 3). As the association of this finding to post-extraction ghost appearance is very close (Figure 7), causation may be inferred. In other words, the etiology of ghosts is the incision of the fibrous tissue covering the leads whose outer part remains in the right cavity after the catheter extraction because it is fixed to the cardiac wall by FAC visualized in vivo by ICE for the first time (but in only 50% of our patients with Ghost possibly because tenuous adhesion are more difficult to be imaged) (Table 2).

Hence, the nature of these post-extraction masses must be mainly fibrotic, but with a certain component of fresh thrombus possibly further induced by the tearing of the fibrotic tissue during the extraction. This theory is mainly confirmed by our follow-up study of the ghost masses that mostly remained unchanged at 1 month while a minority disappeared due to lysis or embolism or both. A rapid (a couple of weeks) clot dissolving process, in fact, takes place when the thrombogenic stimulus was removed [14]. In our view the last hypothesis (lysis and microembolism) is the most realistic as no major episode of clinically evident embolism occurred in the group of patients whose mass had disappeared at the follow-up. In our study, we did not monitor microembolism, but as reported in the literature there are several biomarkers that can be used in monitoring thrombo-microembolism [25]. Such monitoring is certainly warranted in our ghost patients and must be done in the future.

### 4.3. Septic Risk Connected to Ghost

Our data do not support the septic nature of ghost masses even if a case per case judgment must be made (Table 3). We have demonstrated that ghosts are associated with-and thus originate from-remnants of the outer part of the lead fibrous sheath connected to the cardiac wall by other fibrous tissue. In septic patients like ours the catheter with its central canal is the core of infection and that is what has been extracted [26]. Hence, the remnant of tissue encapsulating the lead (ghost) in the cardiac chamber may have a minor or no bacterial load and could be more accessible to the white blood cells. Our data support this since we found no septic complication except in one patients who died of ARDS (acute respiratory distress syndrome) probably related to septic embolism (Table 4). Blood cultures were always negative, except in one patient who recovered (Table 3). Finally, the prognosis was fairly good and was not altered by new infective episodes.

### 4.4. Previous Studies

Only 3 studies [3,4,5] have previously dealt with ghosts but suffered from the major limitations that no study was made of lead fibrosis-that is the “Rosetta stone” to understand the ghost origin. Ignoring this major confounder makes their conclusion that the ghosts are related to the endocarditis a possibly spurious association [27]; this spurious association could perhaps be explained by the fact that lead fibrosis is correlated to both ghosts and also, weakly, perhaps favoring bacterial seeding [24], to vegetations. This spurious association of ghosts to vegetations was also found, even if only as a trend, in our study (Table 3). In addition, two of the three previous studies [4,5] was either retrospective or not performed immediately after lead extractions or employed an imaging technique (like TTE or TEE), that is inferior to ICE [13] and that did not permit appropriate ghost visualization. This may limit their conclusions and explain along with the technique used for extraction (laser excimer sheath) that vaporizes fibrous tissue, the very low detection rate of ghosts in these previous studies.

### 4.5. Clinical Implication

Our data show that the fear of a septic origin of ghosts and of resulting dreadful septic implications is unwarranted; this is confirmed by a recent study [5]. We consider that indiscriminate multi-antibiotic treatment after ghosts is not necessary and can indeed provoke major complications in more susceptible subjects (see our patient with Fabry disease who died from severe antibiotics-related liver and renal impairment) (Table 4) [28]. It may be considered in cases of positive blood cultures post-extraction. Indirect confirmation (TEE follow-up) allows us to affirm that a variable clotting component was present in the 37% of our patients with ghosts so a short term anticoagulation therapy after extraction is warranted in order to reduce symptomatic or asymptomatic pulmonary embolism episodes.

FC and TL assessment is a very useful marker of lead status, with a potentially important clinical impact. In cases of lead encapsulation, close surveillance to identify lead complications is necessary [9] and antibiotic prophylaxis should be instituted to prevent patient exposure to transient bacteremia, as an encapsulated lead is more prone to microorganism seeding [24,29].

### 4.6. Limitations

This study has limited sample size, precluding a more thorough statistical analysis, especially through multivariate modeling of the factors associated with the two major outcomes (ghost and lead fibrosis).

The ICE evaluation of a TL could be compared only qualitatively with what remained of the fibrotic capsule after extraction. Furthermore, no systematic assessment was made of how intact the insulation of the lead was.

Histopathological analysis of the ghost, sporadically reported in the literature [30], was not performed. However, we have an indirect element on which to infer ghost composition.

Ghost formation in the anonymous veins was not explored with ICE.

## 5. Conclusions

ICE is able to evaluate fibrosis involving the lead, as indicated by TL and FAC. Lead fibrosis, but not infection plays a major role in ghosts’ origin; FAC is probably a less specific predictor than TL, because more tenuous strands/adherence are more difficult to visualize. On the basis of our results, ghosts are mainly benign remnants of fibrotic lead capsule, cut during extraction, but retained inside the heart by FAC. Larger studies are needed to confirm these first results.

## Figures and Tables

**Table 1:** Demographic and clinical data.

Demographic and clinical variables	Data
Enrolled patients, #	40
Age, years	75 ± 11
Gender	
Males, #	33
Females, #	7
BMI	27 ± 5
Chronic RF, # (%)	15 (37%)
Diabetes, # (%)	14 (35%)
Hypertension, # (%)	35 (87%)
COPD, # (%)	9 (22%)
LVEF,%	46
GFR, mL/min/1.73 m^2^	73 ± 23
Number of leads removed during each procedure	2 ± 0.7
Atrial leads removed overall, # (%)	33 (85%)
Ventricular leads removed overall, # (%)	36 (95%)
CS leads removed overall, # (%)	9 (23%)
Time from first implantation (m)	113 ± 70
Time from last operation (m)	28 ± 28

# —number of patients; BMI—body mass index; COPD—chronic obstructive pulmonary disease; GFR—glomerular filtration rate; LVEF—left ventricular ejection fraction; RF—renal failure; CS—coronary sinus; m—number of months.

**Table 2:** Factors modulating ICE signs of lead fibrosis (univariate analysis).

Variables		No TL/FAC Group (*n* = 15)	TL/FAC Group (*n* = 25)	Odds Ratio (95% CI)	*p*-Value
**Age**		78 ± 8	74 ± 12	-	ns
Sex, #pt (%)	male	12 (80.0)	21 (84.0)		
	female	3 (20.0)	4 (16.0)		ns
BMI		25.94 ± 3.30	27.92 ± 5.91	-	ns
Diabetes, #pt (%)	-	12 (80.0)	14 (56.0)	1	
	+	3 (20.0)	11 (44.0)	3.14 (0.70–14)	=0.12
EF (%)		47 ± 12	45 ± 11	-	ns
CRF, #pt (%)	-	9 (60.0)	16 (64.0)	1	
	+	6 (40.0)	9 (36.0)	0.8 (0.22–3.1)	ns
Pocket culture positive, #pt (%)	-	0 (0)	10 (40)	1	
	+	15(100)	15 (60)	0.04(0.002–0.9)	=0.04
Local device infection, #pt (%)	-	0 (0)	7 (28)	1	
	+	15 (100)	18 (72)	0.07(0.004–1.5)	=0.09
Veg by ICE, #pt (%)	-	7 (47)	6 (24)	1	
	+	8 (53)	19 (76)	2.7(0.7–10)	ns
CRP (mg/L)		9.07 ± 10.37	17.83 ± 31.06	-	ns
White cells (count)		5.77 ± 1.51	7.52 ± 2.40	-	<0.05
ESR (mm/h)		29.33 ± 22	29.88 ± 22.47	-	ns
Number of leads		2.22 ± 0.6	2.13 ± 0.3	-	ns
Implantation time (months)		77 ± 61	134 ± 67	-	<0.05

# pts—number of patients; TL—thickened lead; FAC—lead fibrotic attachment to the cardiac wall; BMI—body mass index; CI—confidence interval; CRF—chronic renal failure; Veg—vegetations; CRF—chronic renal failure; EF—ejection fraction; m—months; CRP—C reactive protein; ESR—erythrocytes sedimentation rate; OR—odds ratio; -—factor absent; +—factor present.

**Table 3:** Ghost predictors (univariate analysis).

Variables		No Ghost Group (*n* = 16)	Ghost Group (*n* = 24)	OR (95% CI)	*p*-Value
Patient-related factors					
Age		79 ± 9	73± 12	-	ns
Sex, # (%)	male	13 (81.2%)	20 (83.3%)		
	female	3 (18.8%)	4 (16.7%)		ns
BMI		25.91 ± 3.19	28.03 ± 6.01	-	ns
Duke crit, # (%)	Possible endc	5 (31.2%)	5 (20.8%)	-	
	Definite endc (2 criteria)	9 (56.2%)	13 (54.2%)	-	
	Definite endc (>2 criteria)	2 (12.5%)	6 (25%)	-	ns
LVEF		47 ± 12	45 ± 12	-	ns
CRF, # (%)	-	10 (62.5%)	15 (62.5%)	1	
	+	6 (37.5%)	9 (37.5%)	1.2(0.27–3.22)	ns
Diabetes, # (%)	-	13 (81%)	13 (54%)	1	
	+	3 (18%)	11 (46%)	3.7(0.82–16.3)	=0.08
Infectious factors					
CRP (mg/L)		9.26 ± 10.04	18.06 ± 31.7	-	ns
WBC (count*1000)		5.98 ± 1.68	7.45 ± 2.43	-	<0.05
ESR (mm/h)		29.50 ± 21.27	29.79 ± 22.9	-	ns
Fever, # (%)	-	12 (75%)	18 (75%)	1	
	+	4 (25%)	6 (25%)	1.0(0.2–4.32)	ns
Local device infection, # (%)	-	0 (0%)	7 (29%)	1	
	+	16 (100%)	17 (71%)	0.07(0.003–1.78)	ns
Pocket culture positive, # (%)	-	0 (0%)	10 (42%)	1	
	+	16 (100%)	14 (58.3%)	0.04(0.002–.078)	<0.05
Lead tip culture* positive, #(%)	-	16 (100%)	19 (79%)	1	
	+	0 (0%)	5 (21%)	9.3(0.5–181)	ns
BC pre-extraction positive, #(%)	-	11 (69%)	16 (67%)	1	
	+	5 (31%)	8 (33%)	2.1(0.08–54.9)	ns
BC post-extraction positive, #(%)	-	16 (100%)	23 (96%)	1	
	+	0 (0%)	1 (4.2%)	2.1(0.08–54.9)	ns
Endocarditis Vegetation by ICE, # (%)	-	8 (50%)	5 (21%)	1	
	+	8 (50%)	19 (79%)	2.7(0.90–8.1)	=0.073
Lead-related factors					
Number of leads		2.25 ± 0.93	2.08 ± 0.58		ns
lead implantation time (m)		78 ± 59	135 ± 68		<0.05
Thickened lead, # (%)	-	15 (94%)	0 (0%)	1	
	+	1 (6.2%)	24 (100%)	506(19–13,324)	<0.0001
FAC by ICE, # (%)	-	16 (100%)	12 (50%)	1	
	+	0 (0%)	12 (50%)	33(1.8–612)	=0.002
Type of device, # (%)	PM	8 (37%)	14 (58%)		
	ICD	10 (62%)	10 (42%)		ns

# pts—number of patients; *—lead tip culture positive only, no device pocket infection; BMI—body mass index; Endc—endocarditis; CRF—chronic renal failure; CRP—C reactive protein; WBC—white blood cells; ESR—erythrocytes sedimentation rate; BC—blood culture; LVEF—left ventricular ejection fraction; ICD—implantable cardioverter defibrillator; ICE—intracardiac echocardiography; m—months; PM—pacemaker; -—factor absent; +—factor present;

**Table 4:** Main features in patients who died.

Pt No	Age(years)/Gender	Type of Device	Heart Disease	LV EF	Complication of Extraction	Ghost Presence	Ghost Max Length (mm)	Days after Explantation, Cause of Death
2	76/M	ICD	Ischemic DCM	27%	-	no	-	62, Acute HF
4	78/M	ICD	Ischemic DCM	25%	-	yes	8	20, ARDS
12	49/F	PM	Infiltrative CM	45%	-	yes	7	35, MOF
24	81/M	ICD	Ischemic DCM	30%	-	no	-	81, Acute HF

ICD—implantable cardioverter defibrillator; PM—pacemaker; DCM—dilated cardiomyopathy; CM—cardiomyopathy (Fabry disease related); HF—heart failure; ARDS—acute respiratory distress syndrome; MOF—multiple organ failure (liver and kidney) triggered by multi-antibiotic treatment

## Supplementary Materials

The following are available online at https://www.mdpi.com/2077-0383/9/8/2571/s1, Figure S1: Intra and inter observer variability of lead thickness measurement. The limits of agreement are narrow for both the intra and inter-observer graph (within 1 mm) yielding a coefficient of repeatability from duplicate measurement at 0.74 mm and 0.77 mm, respectively, Video S1: Linked to Figure 1. See Figure 1 for explanation, Video S2: Linked to Figure 3. See Figure 3 for explanation, Video S3: Linked to Figure 5. See Figure 5 for explanation.

## References

[1] Sohail M.R., Uslan D.Z., Khan A.H., Friedman P.A., Hayes D.L., Wilson W.R., Steckelberg J.M., Jenkins S.M., Baddour L.M. (2008). Infective endocarditis complicating permanent pacemaker and implantable cardioverter-defibrillator infection. Mayo Clin. Proc..

[2] Wazni O., Epstein L.M., Carrillo R.G., Love C., Adler S.W., Riggio D.W., Karim S.S., Bashir J., Geenspon A.J., DiMarco J.P. (2010). Lead extraction in the contemporary setting: The LExICon study: An observational retrospective study of consecutive laser lead extractions. J. Am. Coll. Cardiol..

[3] Le Dolley Y., Thuny F., Mancini J., Casalta J.P., Riberi A., Gouriet F., Bastard E., Ansaldi S., Franceschi F., Renard S. (2010). Diagnosis of cardiac device-related infective endocarditis after device removal. JACC Cardiovasc. Imaging.

[4] Narducci M.L., Di Monaco A., Pelargonio G., Leoncini E., Boccia S., Mollo R., Perna F., Bencardino G., Pennestrì F., Scoppettuolo G. (2016). Presence of ‘ghosts’ and mortality after transvenous lead extraction. Ep Eur..

[5] Poterała M., Kutarski A., Brzozowski W., Tomaszewski M., Gromadziński L., Tomaszewski A. (2020). Echocardiographic assessment of residuals after transvenous intracardiac lead extraction. Int J. Cardiovasc. Imaging.

[6] Esposito M., Kennergren C., Holmstrom N., Nilsson S., Eckerdal J., Thomsen P. (2002). Morphologic and immunohistochemical observations of tissues surrounding retrieved transvenous pacemaker leads. J. Biomed. Mater. Res..

[7] Candinas R., Duru F., Schneider J., Luscher T.F., Stokes K. (1999). Postmortem analysis of encapsulation around long-term ventricular endocardial pacing leads. Mayo Clin. Proc..

[8] Stokes K., Anderson J., McVenes R., McClay C. (1995). The encapsulation of polyurethane-insulated transvenous cardiac pacemaker leads. Cardiovasc. Pathol..

[9] Kolodzinska A., Kutarski A., Koperski L., Grabowski M., Malecka B., Opolski G. (2012). Differences in encapsulating lead tissue in patients who underwent transvenous lead removal. Ep Eur..

[10] Becker A.E., Becker M.J., Claudon D.G., Edwards J.E. (1972). Surface thrombosis and fibrous encapsulation of intravenous pacemaker catheter electrode. Circulation.

[11] Becker A.E., Becker M.J., Martin F.H., Edwards J.E. (1972). Bland Thrombosis and Infection in Relation to Intracardiac Catheter. Circulation.

[12] Rizzello V., Dello Russo A., Casella M., Biddau R. (2008). Residual fibrous tissue floating in the right atrium after percutaneous pacemaker lead extraction: An unusual complication early detected by intracardiac echocardiography. Int. J. Cardiol..

[13] Narducci M.L., Pelargonio G., Russo E., Marinaccio L., Di Monaco A., Perna F., Bencardino G., Casella M., Di Biase L., Santangeli P. (2013). Usefulness of Intracardiac Echocardiography for the Diagnosis of Cardiovascular Implantable Electronic Device–Related Endocarditis. J. Am. Coll. Cardiol..

[14] Wessler S., Freiman D.G., Ballon J.D., Katz J.H., Wolff R., Wolf E. (1961). Experimental Pulmonary Embolism with Serum-Induced Thrombi. Am. J. Pathol..

[15] Durack D.T., Lukes A.S., Bright D.K., Service D.E. (1994). New criteria for diagnosis of infective endocarditis: Utilization of specific echocardiographic findings. Am. J. Med..

[16] Klug D., Lacroix D., Savoye C., Goullard L., Grandmougin D., Hennequin J.L., Kacet S., Lekieffre J. (1997). Systemic infection related to endocarditis on pacemaker leads: Clinical presentation and management. Circulation.

[17] Bongiorni M.G., Soldati E., Zucchelli G., Di Cori A., Segreti L., De Lucia R., Solarino G., Balbarini A., Marzilli M., Mariani M. (2008). Transvenous removal of pacing and implantable cardiac defibrillating leads using single sheath mechanical dilatation and multiple venous approaches: High success rate and safety in more than 2000 leads. Eur. Hear. J..

[18] Victor F., De Place C., Camus C., Le Breton H., Leclercq C., Pavin D., Mabo P., Daubert C. (1999). Pacemaker lead infection: Echocardiographic features, management, and outcome. Heart.

[19] Sanfilippo A.J., Picard M.H., Newell J.B., Rosas E., Davidoff R., Thomas J.D., Weyman A.E. (1991). Echocardiographic assessment of patients with infectious endocarditis: Prediction of risk for complications. J. Am. Coll. Cardiol..

[20] Altman D.G. (1991). Practical Statistics for Medical Research.

[21] Bland J.M., Altman D.G. (1986). Statistical methods for assessing agreement between two methods of clinical measurement. Lancet.

[22] Narducci M.L., Di Monaco A., Pelargonio G., Leoncini E., Boccia S., Mollo R., Perna F., Bencardino G., Pennestrì F., Scoppettuolo G. (2017). Ghostbusters should come back to lead extraction arena in order to fight with ghosts: Author’s reply. Ep Eur..

[23] Koneru J.N., Ellenbogen K.A. (2013). Detection of transvenous pacemaker and ICD lead vegetations: The ICE cold facts. J. Am. Coll. Cardiol..

[24] Caiati C., Pollice P., Lepera M.E., Favale S. (2019). Pacemaker Lead Endocarditis Investigated with Intracardiac Echocardiography: Factors Modulating the Size of Vegetations and Larger Vegetation Embolic Risk during Lead Extraction. Antibiotics.

[25] Zhang M., Zhang Y., Pang W., Zhai Z., Wang C. (2019). Circulating biomarkers in chronic thromboembolic pulmonary hypertension. Pulm. Circ..

[26] Bongiorni M.G., Di Cori A., Soldati E., Zucchelli G., Segreti L., Solarino G., De Lucia R., Sergi D. (2009). [Iatrogenic risk of permanent pacemaker and defibrillator implantation]. G Ital Cardiol (Rome).

[27] Frost J. (2019). Introduction to Statistics: An Intuitive Guide.

[28] Blumenthal K.G., Peter J.G., Trubiano J.A., Phillips E.J. (2019). Antibiotic allergy. Lancet.

[29] Freedman L.R. (1987). The pathogenesis of infective endocarditis. J. Antimicrob. Chemother..

[30] Andreas M., Wiedemann D., Kocher A., Khazen C. (2013). Materialization of ghosts: Severe intracardiac masses after pacemaker lead extraction requiring immediate surgical intervention. Heart Rhythm.

