# Supplementary material for: A Novel Clinical Perspective on New Masses after Lead Extraction (Ghosts) by Means of Intracardiac Echocardiography
Source: J Clin Med. 2020 Aug 8;9(8):2571. doi: 10.3390/jcm9082571 (PMC7465795; doi:10.3390/jcm9082571)
Supplement: Supplementary file 1 [file jcm-09-02571-s001.pdf]

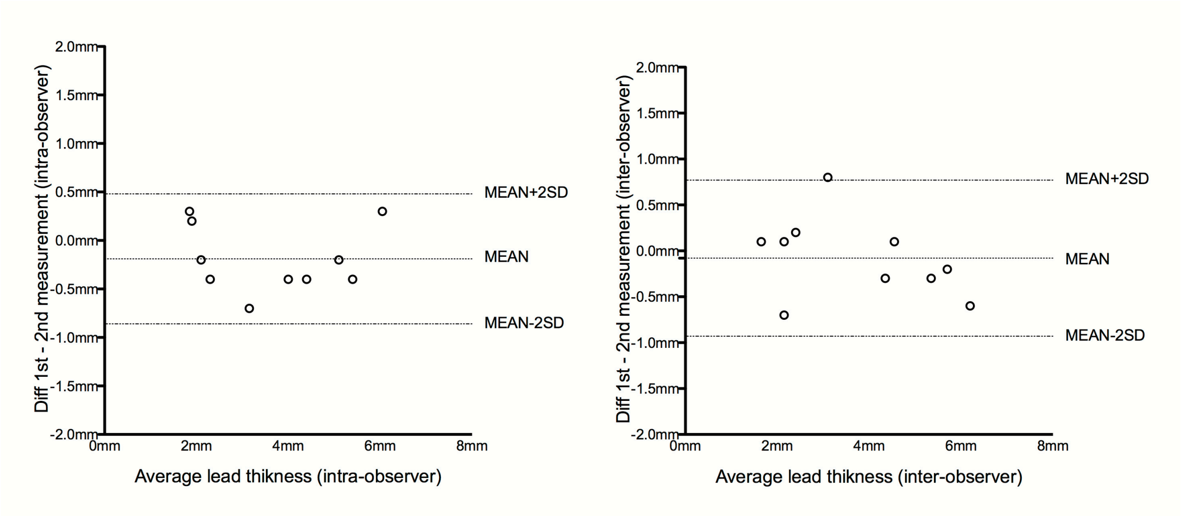

Supplemental Figure 1. Intra and inter observer variability of lead thickness measurement. The limits of agreement are narrow for both the intra and inter-observer graph (within 1 mm) yielding a coefficient of repeatability from duplicate measurement at 0.74 mm and 0.77 mm respectively.

VIDEO LEGENDS.

Video **1**. Linked to Figure 1. See Figure 1 for explanation.

Video **2**. Linked to Figure 3. See Figure 3 for explanation.

Video **3**. Linked to Figure 5. See Figure 5 for explanation.
